# The Long Noncoding RNA *RPS10P2-AS1* Is Implicated in Autism Spectrum Disorder Risk and Modulates Gene Expression in Human Neuronal Progenitor Cells

**DOI:** 10.3389/fgene.2019.00970

**Published:** 2019-10-15

**Authors:** Stephanie M. Bilinovich, Kristy Lewis, Nicole Grepo, Daniel B. Campbell

**Affiliations:** ^1^Department of Pediatrics and Human Development, Michigan State University, Grand Rapids, MI, United States; ^2^Center for Gene Therapy, City of Hope, Duarte, CA, United States

**Keywords:** epigenetics, brain, development, autism, lncRNA, noncoding RNA, genetics, environment

## Abstract

Most of the genetic risk for autism spectrum disorder (ASD) is inherited as common genetic variants, although some rare mutations have been identified in individuals with ASD. Common genetic variants are most parsimoniously identified by genome wide association studies. Genome wide association studies have identified several genetic loci with genome wide association with ASD. However, genome wide association studies only identify regions of the genome associated with phenotypic traits. Identification of the functional elements requires additional experimental evidence. Here, we demonstrate that a genome wide association study locus for ASD on chromosome 20p12.1, rs4141463, implicates a noncoding RNA as a functional element. Although rs4141463 lies within an intron of the protein-coding *MACROD2* (MACRO domain containing 2) gene, expression of *MACROD2* is neither altered in postmortem temporal cortex of individuals with ASD nor correlated with rs4141463 genotype. Our bioinformatics approaches revealed a noncoding RNA transcript near the autism susceptibility signal, *RPS10P2-AS1* (ribosomal protein S10 pseudogene 2 anti-sense 1). In a panel of 15 human tissues, *RPS10P2-AS1* was expressed at higher levels than the protein-coding *MACROD2* in both fetal temporal cortex and adult peripheral blood. In postmortem temporal cortex, expression of *RPS10P2-AS1* was increased 7-fold in individuals with ASD (P = 0.02) and increased 8-fold in individuals with the ASD-associated rs4141463 genotype (P = 0.01). Further, *RPS10P2-AS1* expression was increased in human neural progenitor cells exposed to model air pollutants, indicating that both genetic and environmental factors that contribute to ASD increased *RPS10P2-AS1* expression. Overexpression of *RPS10P2-AS1* in human neural progenitor cells indicated substantial changes in neuronal gene expression. These data indicate that genome-wide significant associations with ASD implicate long noncoding RNAs. Because long noncoding RNAs are more abundant in human brain than protein-coding RNAs, this class of molecules is likely to contribute to ASD risk.

## Introduction

Autism spectrum disorder (ASD) is a neurodevelopmental disorder that is characterized by deficits in social communication and behavioral flexibility (Levitt and Campbell, 2009). ASD symptoms begin by age 3, last throughout the lifespan, and impact 1.5% of the population (Beversdorf and MISSOURI AUTISM SUMMIT CONSORTIUM, 2016). The causes of ASD are an active area of research. Although *de novo* loss of function mutations have been highlighted in the genetics of ASD, it is clear that the majority of ASD genetic influences are from common genetic variants of relatively small effect (Anney et al., 2012; Gaugler et al., 2014). Association of common genetic variants is most efficiently identified by genome-wide association studies (GWAS). The first published GWAS for ASD reported genome-wide significant association (P < 5 × 10^-8^) of genetic markers on chromosome 5p14.1 (Wang et al., 2009). These markers mapped between two protein-coding genes known to be involved in brain development. However, we recently reported that the functional element implicated by the chromosome 5p14.1 genetic markers was a long noncoding RNA, Moesin Pseudogene 1 Anti-Sense (*MSNP1AS*) (Kerin et al., 2012). The expression of *MSNP1AS* was increased in postmortem cerebral cortex of both individuals with ASD and individuals with the genotypes associated with ASD; the expression of neither of the neighboring chromosome 5p14.1 protein-coding genes was altered in ASD or correlated with the genotypes associated with ASD (Kerin et al., 2012). We therefore concluded that *MSNP1AS* was the functional element revealed by the chromosome 5p14.1 ASD genome-wide association peak (Kerin et al., 2012). We later found that over-expression of *MSNP1AS* in human neural progenitor cells altered neuronal morphology and expression of genes involved in two biological processes: protein synthesis and chromatin remodeling (DeWitt et al., 2016a). Similarly, transcriptional gene silencing of *MSNP1AS* in human neural progenitor cells altered expression of genes involved in chromatin remodeling and immune response (DeWitt et al., 2016b). The biological processes implicated by altered *MSNP1AS* expression in human neural progenitor cells are reminiscent of those implicated by rare *de novo* loss-of-function mutations, and thus suggest a convergent biology of ASD implicated by divergent genetic association approaches (DeWitt et al., 2016a; DeWitt et al., 2016b).

The second published GWAS for ASD reported genome-wide significant association of a single genetic marker, rs4141463, on chromosome 20p12.1 (Anney et al., 2010). The rs4141463 marker lies within an intron of the protein-coding gene MACRO domain containing 2 (*MACROD2*). Based on our previous findings of implication of the long noncoding RNA *MSNP1AS* by the chromosome 5p14.1 genome-wide association instead of the neighboring protein-coding genes, we hypothesized that the chromosome 20p12.1 genome-wide association might also implicate a genetic element other than protein-coding *MACROD2* gene. Bioinformatics approaches indicated the presence of a long noncoding RNA, Ribosomal Protein S10 Pseudogene 2 Anti-Sense 1 (*RPS10P2-AS1*) at the site of the rs4141463 association with ASD. In this report, we provide evidence that *RPS10P2-AS1* is the functional element revealed by the chromosome 20p12.1 ASD genome-wide significant association signal and begin to determine the functions of *RPS10P2-AS1* in human neural progenitor cells.

## Materials and Methods

Postmortem brain samples: Postmortem brain samples from 10 individuals with ASD and 10 age- and gender-matched controls were obtained from the Autism Tissue Program, now known as Autism BrainNet. All samples were Brodmann Area 22, corresponding to the superior temporal gyrus. Specific information about age- and gender-matching are listed in **Table S1**. Genotypes at the rs4141463 locus for each of the brain samples was determined by PCR amplification and Sanger sequencing.

Cell Culture: The human neuronal progenitor cell ReNcell CX was used in this study as the cells differentiate into cortical projection neurons (EMD Millipore, Darmstadt, Germany). ReNcell CX cells were maintained in tissue culture flasks at 37°C and 5% CO2 in ReNcell NSC Maintenance Medium supplemented with FGF and EGF in laminin-coated flasks. Diesel particulate matter was purchased from Sigma, serially diluted in a small volume of dimethyl sulfoxide (Sigma), and added to the ReNcell medium for 24 h prior to harvest.

Over-expression transfection: To determine the impact of *RPS10P2-AS1* over expression on gene expression in human neuronal cells, *RPS10P2-AS1* was PCR-amplified using the KOD Xtreme Hot Start kit (Novagen, Madison, WI, USA). PCR product was first cloned into pSTBlue-1 then sub-cloned into pIRES2-AcGFP1 and pIRES2-DsRed2 vectors (Clonetech, Mountain View, CA, USA) using restriction sites to create sense and antisense clones. PCR product and vectors were sequence verified at multiple points during the cloning process. Cells were then grown to ∼70% confluency and cultured 24 h prior to experimentation. Cells were transfected with *RPS10P2-AS1* and the reverse complement control (*RPS10P2*) over-expression vectors using Amaxa Nucleofector kit (Lonza, Basel, Switzerland). Empty pIRES2 vectors were used as controls. We optimized the transfection experiments by manipulating the mass of plasmid transfected (1 mg) and the duration of transfection (Amaxa program T-16) to achieve a consistent 8-fold increase in *RPS10P2-AS1* in ReNcell CX cells after 24 h. Experiments were harvested at 24 h post-transfection and were performed in quadruplicate. Prior to RNA sequencing, qPCR was used to confirm over-expression of *RPS10P2-AS1* by 7.7- to 8.3-fold.

RNA Isolation: Experimental cells were harvested and pelleted by centrifugation. Cell pellets were homogenized using Qiashredder spin columns (Qiagen, Valencia, CA, USA) and then total RNA was extracted using Qiagen RNeasy kit according to the manufacturer’s protocol. Additionally, total RNAs (Biochain, Newark, CA, USA) were purchased to look at expression in tissues of other areas of the human body. Total RNAs purchased include: Adult Normal Tissue Brain Temporal Lobe (R1234078-50), Adult Normal Tissue Brain Cerebellum (R1234039-50), Adult Normal Tissue Brain Frontal Lobe (R1234051-50), Adult Normal Tissue Brain Occipital Lobe (R1234062-50), Adult Normal Tissue Spinal Cord (R1234234-50), Adult Normal Tissue Peripheral Blood Leukocyte (R1234148-10), Adult Normal Tissue Heart (R1234122-50), Adult Normal Tissue Lung (R1234152-50), Adult Normal Tissue Kidney (R1234142-50), Adult Normal Tissue Skin (R1234218-50), Fetal Normal Tissue Brain Temporal Lobe (R1244078-50), Fetal Normal Tissue Brain Frontal Lobe (R1244051-50), Fetal Normal Tissue Heart (R1244122-50), Fetal Normal Tissue Lung (R1244152-50) and Fetal Normal Tissue Kidney (R1244142-10).

Quantitative RT-PCR: RNA extracted samples were converted to cDNA using Life Technologies Superscript III RT-PCR kit (Life Technologies, Carlsbad, CA, USA). Then qPCR was performed on a Life Technologies StepOnePlus PCR System using the following TaqMan gene expression assays: RPS10 (Hs01693877_s1), RPS10P2-AS1 (AI1RVBK), GAPDH (Hs9999905_m1) and POL2RA (Hs00172187_m1). GAPDH and POLR2A serve as housekeeping gene controls. Data was analyzed *via* StepOnePlus software v2.3.

RNA-seq: RNA-seq was performed in order to determine which genes would be differentially expressed as a result of *RPS10P2-AS1* over-expression. First total RNA quality was assessed by using a Nanodrop ND-1000 Spectrophotometer (Thermo Fisher Scientific, Waltham, MA, USA) to look at 260/280 absorbance ratios then using an Agilent Technologies 2200 TapeStation Instrument (Agilent Technologies, Santa Clara, CA, USA) to determine RNA integrity numbers. Next cDNA libraries were constructed from RNA extracted from cell pellets with the Illumina Truseq Stranded Total RNA Sample Preparation kit and sequencing was performed on the HiSeq2000 sequencer to generate 101 bp single-end reads (Illumina, San Diego, CA, USA). The average number of reads per sample run was 36 million reads. Each *RPS10P2-AS1* transfection was performed in quadruplicate. RNA-seq was performed on each of the four transfection trials.

RNA-seq analysis: RNA sequencing analysis was performed as previously described (Wilkinson et al., 2015). Briefly, sample files were run though a quality control pipeline by first removing adapters from raw reads with the Cut Adapt program (Martin, 2011). Then sample files were aligned to available genome builds (UCSC Hg19, Ensembl GrCH37 and Ensembl GrCH38) using Tophat2 (Kim et al., 2013). After alignment, fastq files were run through the FastQC program to further assess quality of files 12. Once samples were mapped differential gene expression analysis was performed using Cuffdiff with corresponding transcriptomes under default settings which compared *RPS10P2-AS1* over-expression vector to empty vector controls at each time point per cell line (Trapnell et al., 2012). A list of significantly differentially expressed genes was entered into the Database for Annotation, Visualization and Integrated Discovery (DAVID) for gene enrichment analysis for each cell line at each time point (Huang et al., 2009). It is important to note that Ensembl builds inherently contain more ncRNAs than the UCSC build, hence, all builds were examined in depth. We report here both P values that are corrected for multiple comparisons (q values) and P values that are uncorrected for multiple comparisons (p values). All discussions of individual genes are those that are q value significant (i.e., significant after correction for multiple comparisons). However, all discussions of downstream gene ontology (DAVID) analyses include all genes that are significant without correction for multiple comparisons.

## Results

The single nucleotide polymorphism (SNP) rs4141463 was assigned genome wide association with ASD through a GWAS 9. The rs4141463 SNP is within an intron of the *MACROD2* gene. However, *MACROD2* spans more than 2 million base pairs (2 Mbp) of chromosome 20, and rs4141463 resides within a particularly large intron that spans more than 500,000 base pairs (500 kb) of DNA sequence. Within the intron of *MACROD2* is the 494 bp long noncoding RNA *RPS10P2-AS1*, which resides just 9 kb from rs4141463. Due to the proximity of both *MACROD2* and *RPS10P2-AS1* to the ASD genome wide significant signal, we decided to test both genes as quantitative trait loci for rs4141463.

**Figure 1:**
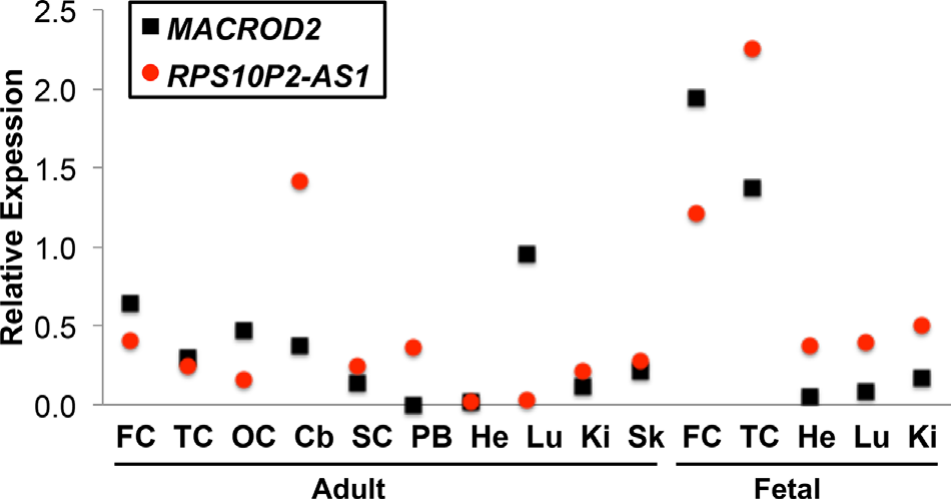
Relative expression of *MACROD2* and *RPS10P2-AS1* in 15 human tissues. Although noncoding RNAs are often expressed at lower levels than protein-coding genes in bulk tissue, *RPS10P2-AS1* is expressed at higher levels than *MACROD2* in all 15 tissue samples tested. *RPS10P2-AS1* is expressed at higher levels than *MACROD2* in adult cerebellum, spinal cord, peripheral blood, kidney and skin and in fetal temporal cortex, heart, lung, and kidney. Each qPCR was performed 4 times and the results averaged. FC, frontal cortex; TC, temporal cortex; OC, occipital cortex; Cb, cerebellum; SC, spinal cord; PB, peripheral blood; He, heart; Lu, lung; Ki, kidney; Sk, skin.

Publicly available gene expression data from the GTex consortium indicated that both *MACROD2* and *RPS10P2-AS1* are expressed at detectable levels in human cerebral cortex (GTEx Consortium, 2015). To get a more detailed map of expression for both genes, we performed qPCR for both *MACROD2* and *RPS10P2-AS1* in a panel of tissues that included several regions of cerebral cortex (**Figure 1**). Our qPCR data indicated that both transcripts are expressed at detectable levels in each of the 10 adult and 5 fetal tissues (**Figure 1**). *RPS10P2-AS1* expression was highest in fetal temporal cortex, fetal frontal cortex and adult cerebellum (**Figure 1**). *MACROD2* expression levels were highest in fetal frontal cortex, fetal temporal cortex, and adult lung (**Figure 1**). Expression of *RPS10P2-AS1* was higher than *MACROD2* in 9 of the 15 tissues, including 4 of the 5 fetal tissues (**Figure 1**).

**Figure 2:**
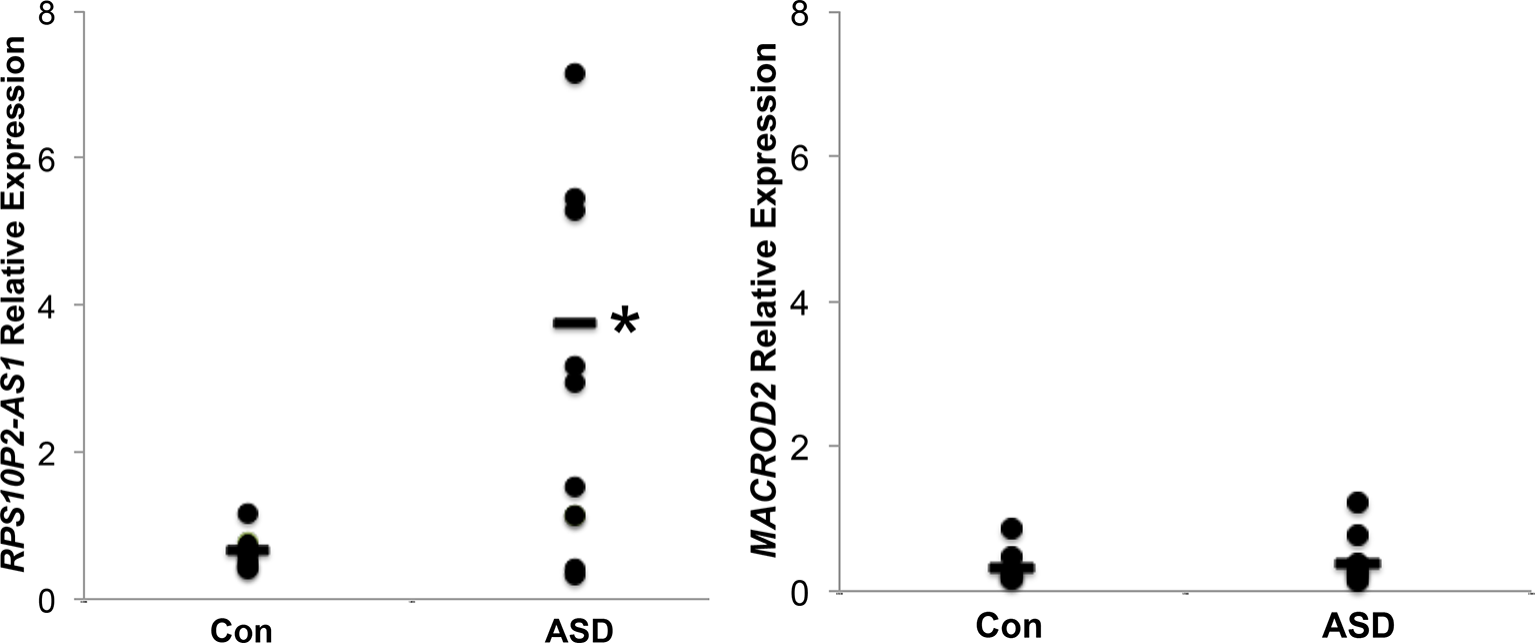
Relative expression of *RPS10P2-AS1* and *MACROD2* in postmortem temporal cortex of 10 individuals with ASD and 10 age- and gender-matched controls. While there is no significant change in *MACROD2* expression in postmortem brain of individuals with ASD, there is a 7-fold increase in *RPS10P2-AS1* in individuals with ASD compared to controls (P < 0.01). Each qPCR was performed 4 times and the results averaged. As should be expected from a disorder with multiple etiologies, half of the ASD brain samples show levels of *RPS10P2-AS1* that is comparable to the controls; however, half of the ASD brain samples show *RPS10P2-AS1* expression substantially higher than any of the controls. * indicates P < 0.01 by Student’s t-test.

We next tested the expression of *MACROD2* and *RPS10P2-AS1* in postmortem temporal cerebral cortex, a region of the brain in which both transcripts are expressed in both fetal and adult tissue (**Figure 1**), of 10 individuals with ASD and 10 non-ASD controls. The expression of *MACROD2* was not significantly different between individuals with ASD and controls (**Figure 2**). In contrast, there was a 7-fold increase in average *RPS10P2-AS1* expression in postmortem temporal cortex of individuals with ASD compared to age- and gender-matched controls (**Figure 2**; P = 0.01). These data indicate that *RPS10P2-AS1* expression, but not *MACROD2* expression, is associated with ASD diagnosis.

**Figure 3:**
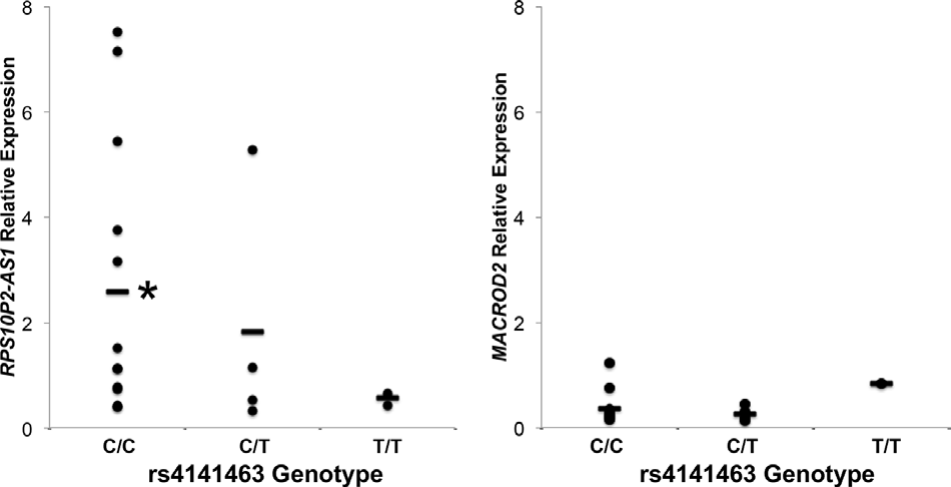
Relative expression of *RPS10P2-AS1* and *MACROD2* by genotype at the rs4141463 locus in postmortem temporal cortex. The common rs4141463 C allele is associated with ASD by GWAS. The C allele is associated with higher expression of *RPS10P2-AS1*, but is not correlated with expression levels of *MACROD2*. N = 4. * indicates P < 0.01 by ANOVA followed by Tukey-Kramer test.

To determine the association between rs4141463 genotype and expression of *MACROD2* and *RPS10P2-AS1* in human temporal cortex, we genotyped rs4141463 in the 20 human temporal cortex samples. The rs4141463 C allele is associated with ASD (Anney et al., 2010). In the postmortem temporal cortex samples, the rs4141463 C/C genotype was associated with a significantly higher expression of *RPS10P2-AS1* than the T/T genotype (**Figure 3**). Individuals with the rs4141463 heterozygous genotype C/T had an intermediate expression of *RPS10P2-AS1* (**Figure 3**). In contrast to the significant association of rs4141463 genotype with *RPS10P2-AS1* expression, there was no correlation between genotype and *MACROD2* expression (**Figure 3**). These data indicate that *RPS10P2-AS1* is the functional element revealed by genome wide significant association of rs4141463. GTEx analysis indicates that rs4141463 does not have a significant eQTL with any gene in any tissue. However, it should be noted that superior temporal cerebral cortex is not among the tissues tested in GTEx.

**Figure 4:**
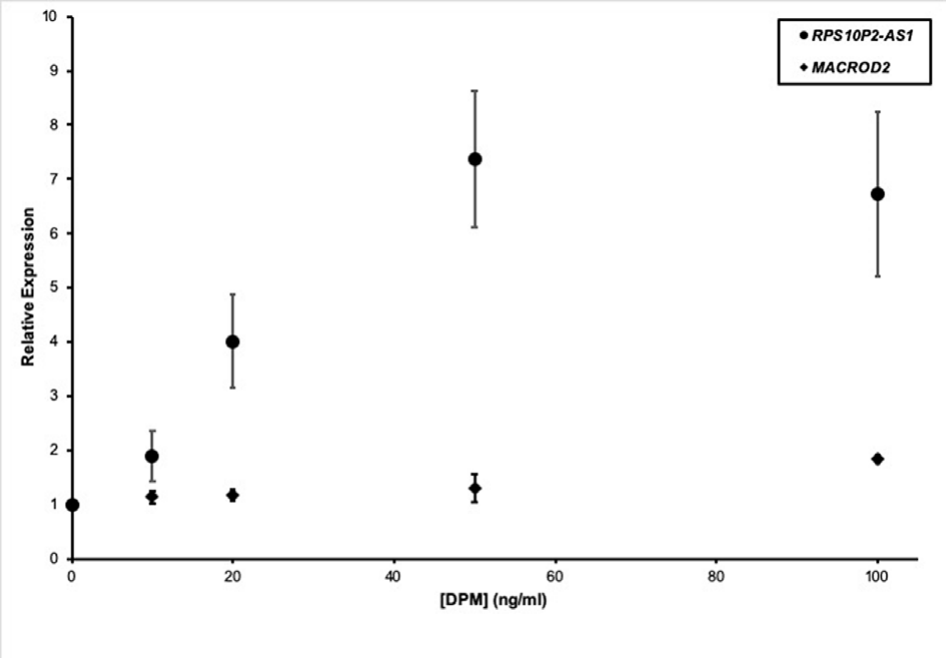
Relative expression of *RPS10P2-AS1* (circles) and *MACROD2* (diamonds) in ReNcell CX human cortical projection neurons following increasing exposure to the model air pollutant diesel particulate matter (DPM). N = 5. * indicates P < 0.05 by ANOVA followed by Dunnett t-test.

In addition to genetic factors, environmental factors can increase ASD risk. For example, near roadway air pollution can double the risk of ASD (Volk et al., 2013). To test the impact of a model air pollutant on *RPS10P2-AS1*, we exposed ReNcell CX human cortical projection neurons to increasing concentrations of diesel particulate matter (DPM). The DPM exposure caused a dose-dependent increase in *RPS10P2-AS1* expression: 10 ng/ml DPM increased *RPS10P2-AS1* by a non-significant 1.9-fold; 20 ng/ml DPM increased *RPS10P2-AS1* by a significant 4-fold; and both 50 and 100 ng/ml DPM caused a significant 7-fold increase in *RPS10P2-AS1* (**Figure 4**). In contrast, exposure of ReNcell CX cells to DPM did not significantly change the expression of the protein-coding gene *MACROD2* (**Figure 4**). These data indicate that exposure of human cortical projection neurons to an ASD-associated environmental factor causes an increase in the noncoding RNA *RPS10P2-AS1* that is on the same scale as the increased expression observed in postmortem cerebral cortex of individuals with ASD. The environmental response of *RPS10P2-AS1* provides further suggestive evidence, in addition to the genetic evidence, that the lncRNA may contribute to ASD risk.

**Figure 5:**
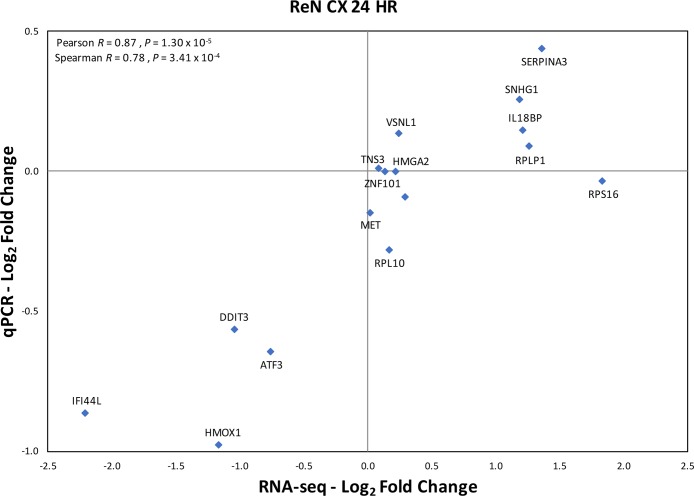
Validation of RNA seq results by qPCR of selected genes in the same samples exposed to increased expression of *RPS10P2-AS1*. N = 4. The qPCR results validate that RNA seq results with Pearson correlation R = 0.87 (P = 1.30 x 10^-5^).

**Figure 6:**
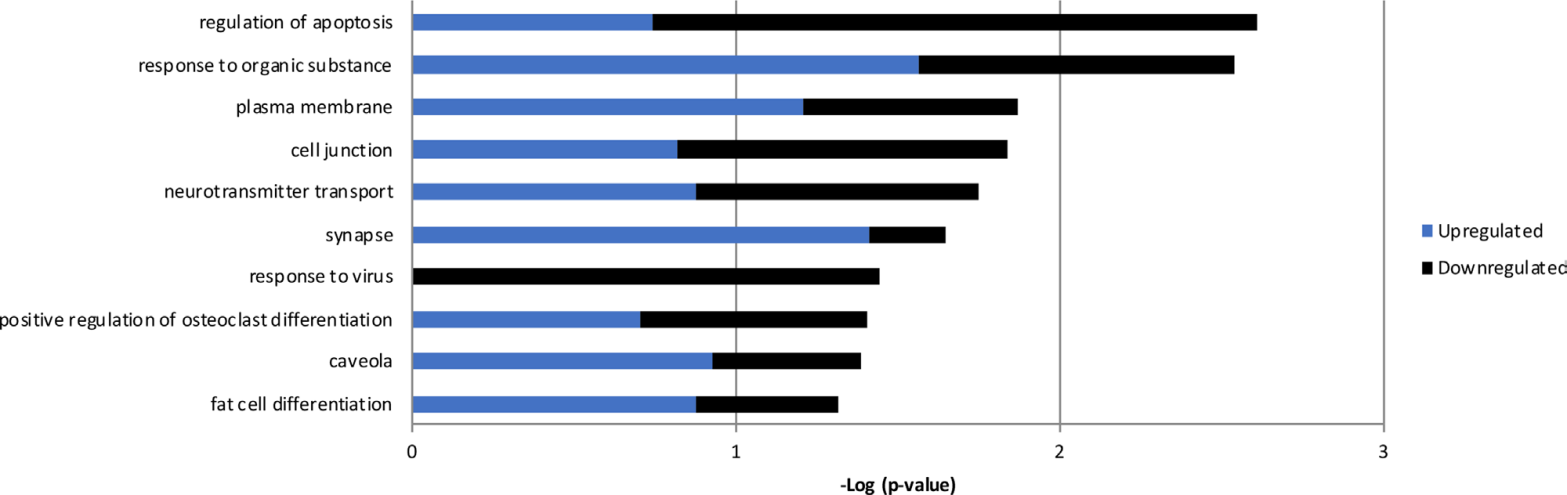
Gene ontology results from the 143 genes with altered expression in RenCell CX cells following over-expression of *RPS10P2-AS1*. Blue indicates enrichment of genes that are upregulated; black indicates enrichment of genes that are downregulated; the combination of enrichment scores is additive. Genes altered by *RPS10P2-AS1* are enriched for genes involved in apoptosis, response to organic substance, neurotransmitter transport and synapse, indicating the *RPS10P2-AS1* alters the ability of a neuron to respond to environmental challenge.

The expression of *RPS10P2-AS1* was increased in postmortem brains of individuals with ASD (**Figure 2**), in postmortem brains of individuals with the ASD-associated rs4141463 C/C genotype (**Figure 3**), and in cultured human projection neurons exposed to DPM (**Figure 4**). To determine the impact of *RPS10P2-AS1* over-expression on gene expression in developing human neurons, we transfected a *RPS10P2-AS1* over-expression construct into ReNcell CX human cortical projection neurons and performed RNA sequencing to determine the impact on gene expression. We calibrated the protocol to over-expression at ∼8-fold increased expression of *RPS10P2-AS1*, similar to the levels observed in postmortem cerebral cortex of individuals with ASD and in ReNcell CX cells exposed to DPM. A total of 143 genes were differentially expressed following *RPS10P2-AS1* over-expression (p < 0.05, **Supplementary Table 2**), with 14 genes differentially expressed following correction for multiple comparisons (q < 0.05, **Supplementary Table 2**). The RNA-seq results were validated by qPCR of selected genes (**Figure 5**) and show a significant correlation between RNA-seq and qPCR (Pearson R = 0.84; P < 1 x 10^-5^). The most significant changes in gene expression involved interferon inducible proteins 6 and 44 (IFI6 and IFI44) and small nuclear RNAs 4-1 and 4-1 (SNU4-1 and SNU4-2) (**Supplementary Table 2**). Gene ontology analysis of the 143 differentially expressed genes (P < 0.05) indicated enrichment of genes involved in apoptosis, response to organic substances, neurotransmitter transport and synapses (**Figure 6**). These data suggest that *RPS10P2-AS1* may play a critical role in neuron development and response to environmental insults.

## Discussion

Our data indicate that the long noncoding RNA *RPS10P2-AS1*, not the protein-coding RNA *MACROD2*, is a functional element revealed by the genome wide significant association of rs4141463 with ASD risk. *RPS10P2-AS1* expression is increased in postmortem temporal cortex of individuals with ASD. Increased *RPS10P2-AS1* expression is correlated with the ASD-associated rs4141463 C allele. *RPS10P2-AS1* expression is increased in human neural progenitor cells following exposure to ASD-associated diesel particulate matter. These data suggest that increased *RPS10P2-AS1* may be a convergent molecular mechanism by which both common genetic variants and environmental factors contribute to increased ASD risk.

Genome wide association studies of ASD have identified several loci with genome wide significant association (Wang et al., 2009; Anney et al., 2010; Autism Spectrum Disorders Working Group of The Psychiatric Genomics Consortium, 2017; Grove et al., 2019). The chromosome 20p12.1 region is one of the few regions that has been implicated multiple times as a recent study identified genome wide significant association of rs71190156, which is 89 kb from rs4141463 and 98 kb from *RPS10P2-AS1* (Autism Spectrum Disorders Working Group of The Psychiatric Genomics Consortium, 2017). Future experiments will be required to determine if association of rs71190156 implicates *RPS10P2-AS1* or a different functional element on chromosome 20p12.1.

The identification of *RPS10P2-AS1* as a functional element revealed by genome wide association of rs4141463 with ASD provides further evidence that long noncoding RNAs contribute to altered brain development. The default assumption has been that association of rs4141463 with ASD implicated *MACROD2*, the protein coding gene within which rs4141463 is located. However, long noncoding RNAs account for half of the RNAs expressed in human brain (Kapranov et al., 2010) and human neural progenitor cells (Hecht et al., 2015). Therefore, it is not surprising that long noncoding RNAs are implicated in multiple neurodevelopmental disorders. We previously identified the long noncoding RNA *MSNP1AS* as a functional element revealed by genome wide significant association with ASD of chromosome 5p14.1 genetic markers (Kerin et al., 2012). The identification of *RPS10P2-AS1* as the functional element revealed by rs4141463 association with ASD provides additional evidence of long noncoding RNA contribution to brain development.

Overexpression of *RPS10P2-AS1* caused significant changes in the expression of 14 genes (q < 0.05) in ReNcell CX cells. The zinc finger transcription factor *ZNF865* was increased in expression more than 16-fold following *RPS10P2-AS1* over-expression. The function of *ZNF865* is unknown, as a PubMed search for ZNF865 yields no results. Overexpression of *RPS10P2-AS1* increased the expression of a mitochondrial enzyme (*RDH13*), two small nuclear RNAs (*RNU4-1* and *RNU4-2*), and two proteins involved in ribosomal function (*RPS16* and *RPLP1*). Genes that decreased in expression following *RPS10P2-AS1* overexpression include interferon-inducible genes (*IFI6*, *MX1* and *IFI44L*), which may play a role in apoptosis, and genes involved in synthesis and shuttling of amino acids (*SLC7A11* and *ASNS*). Gene ontology analysis of the 143 genes with P < 0.05 indicated significant enrichment of genes involved in the regulation of apoptosis, response to organic substances, neurotransmitter transport and synapses. This suggests that *RPS10P2-AS1* may be involved in responses to environmental challenges that impact neuron function.

## Data Availability Statement

The raw data supporting the conclusions of this study can be found in NCBI using accession number PRJNA564157.

## Author Contributions

The experiments were conceived and designed by DC and NG. SB, KL, and NG performed the experiments and data analysis. DC wrote the first draft of the manuscript. All authors contributed to manuscript revision, and read and approved the submitted version.

## Funding

This work was funded by the National Institute of Mental Health (R21MH099504), the National Institute of Environmental Health Sciences (R56 ES 029064), the Michigan State Foundation Discretionary Funds Initiative, and the Spectrum Health-MSU Alliance Corporation.

## Conflict of Interest

The authors declare that the research was conducted in the absence of any commercial or financial relationships that could be construed as a potential conflict of interest.

## Supplementary Material

Supplementary Table 1 Description of donated postmortem brain samples obtained from the Autism Tissue Program, now Autism BrainNet. Each sample was from the superior temporal gyrus, which corresponds to Brodmann area 22. PMI, Post-Mortem Interval.

Supplementary Table 2 List of 143 genes with significant changes (P < 0.05) in expression following over-expression of *RPS10P2-AS1* in ReNcell CX cells.
